# Effect of locally transplanted bone marrow derived mesenchymal stem cells on the lingual filiform and fungiform papillae of ovariectomized albino rat model

**DOI:** 10.1186/s12903-026-08368-6

**Published:** 2026-04-30

**Authors:** Maha El Shahawy, Mona El Deeb

**Affiliations:** 1 Department of Oral Biology, Faculty of Dentistry, Kafrelsheikh University, Elgeish street, Kafr el-sheikh, 33516 Egypt; 2 Oral Biology Department, Faculty of Oral and Dental Medicine, Future University in Egypt, Cairo, 11835 Egypt

**Keywords:** BM-MSCs, Ovariectomized rat, Tongue papillae, Scanning electron microscope, Histopathology

## Abstract

**Background:**

Taste alteration and burning mouth syndrome are major irritating oral disturbances during menopause. Stem cell-based therapy exhibits high regenerative potential in the disturbances of the maxillofacial region. The aim of the current study is to investigate the possible effectiveness of transplanted bone marrow mesenchymal stem cells (BM-MSCs) on the altered filiform and fungiform papillae of the ovariectomized rats.

**Methods:**

Seventy-eight female albino rats were classified into 3 groups; sham operated rat (group I), ovariectomized rat (group II), ovariectomized rats treated with BM-MSC (group III) groups. The animals in each group were further subdivided equally into 2 subgroups (a & b) according to the date of euthanasia (one-and two-months post treatment), (*n* = 13) per subgroup. For statistical analysis, Kruskal Wallis test was done for comparing the 3 experimental groups followed by Post Hoc Dunn´s multiple comparison test for pairwise comparison. The injected BM-MSCs were immunophenotyped for CD90, CD44 and CD45. The bone mineral density (BMD) of rats´ femur was assessed. The tongue specimens were assessed using Hematoxylin and Eosin stain (H&E), Masson trichrome stain, histomorphometry and scanning electron microscope.

**Results:**

Ovariectomized animals revealed low BMD compared to their controls. The lingual papilla depicted abnormal histology and surface structure. In addition, the height, area of connective tissue papillae and collagen area %, were reduced, and the keratin thickness of the inferior lingual epithelium was decreased. The BM-MSC treated group revealed promoted recovery of the regular filiform and fungiform papillary architecture, normal surface structure and histomorphometry.

**Conclusions:**

Transplanted BM-MSCs promoted the recovery of the normal architecture of the lingual papillae of ovariectomized animals and may present a promising therapeutic option in modifying the lingual mucosal alteration caused by ovariectomy.

**Graphical Abstract:**

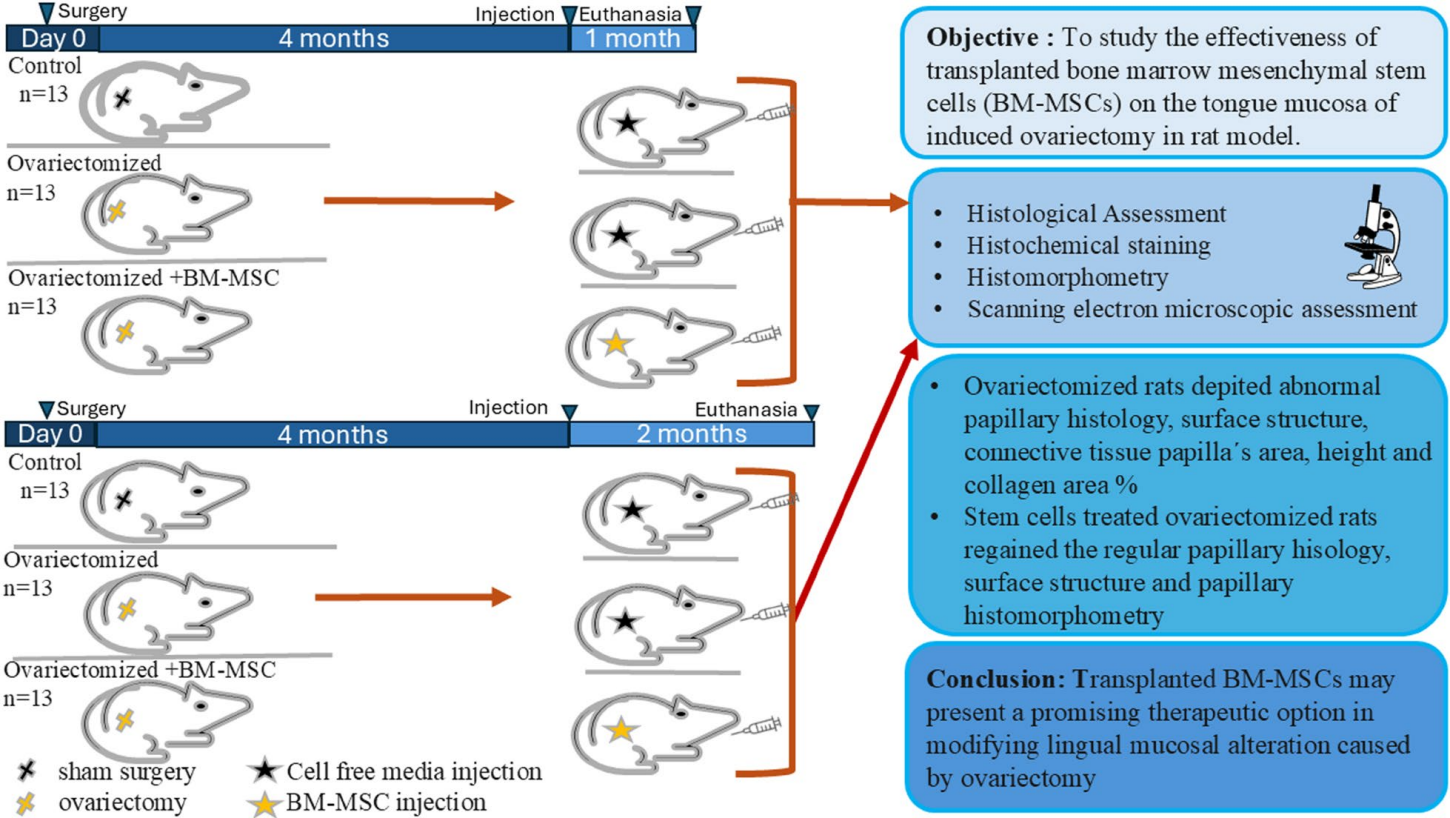

## Highlights

Ovariectomized rats (OVX rats) depicted aberrant histology of the lingual papillae

OVX rats revealed distorted surface structure of the lingual papillae

Local stem cell treatment regained the papilla normal surface structure in OVX rats

Stem cell treatment resumed the lingual papilla regular histology in OVX rats

## Introduction

The tongue is a crucial organ for mastication, speech, swallowing and taste. The dorsal surface of the tongue contains special structures termed papillae. The filiform papillae are the most numerous, covered by keratinized epithelium and do not contain taste buds. The fungiform papillae are situated among the numerous filiform papillae and contain taste buds. The taste buds are multicellular structures that are responsible for perceiving the different taste sensations [1].

Ovaries are the primary source of natural estrogen [2]. In the postmenopausal period, the endogenic estrogen level declines disturbing mostly all body organs [3]. Throughout this period, females experience several physical and morphological alterations including cardiovascular problems, skeletal diseases, hot flushes, and skin atrophy [4]. Definite receptors are responsible for binding to ovarian hormones, allowing them to act on oral mucosal cell cytoplasm or nucleus. The absence of endogenous estrogen triggers alterations in soft tissue cells possessing estrogen receptors [5] including salivary glands and oral mucosa [6]. Therefore, abnormalities in oral tissues occur with the lack of these hormones [7]. Postmenopausal women can develop some common oral symptoms including hyposalivation and altered taste and have high incidence of burning mouth syndrome with burning sensation especially localized in the tongue [8–12]. In addition, postmenopausal women develop mucosal atrophy, decreased flexibility of the oral mucosa and increased liability to oral ulceration while using removal restorations in the coexisted hyposalivation condition. As oral health is closely related to general health and can impact mental and physical health, therefore, it is crucial to consider oral changes in line with other systemic alterations in menopausal women for proper oral health and lifestyle [8].

Ovariectomized animals are a good model to mimic human loss of ovarian hormones. Therefore, various studies evaluated different systems and functions in ovariectomized animals including bone, salivary glands, skin, hepatocytes, central nervous system, immune and vascular functions [12–18]. In addition, ovariectomized animal models have been used to assess the pathophysiology of the burning mouth syndrome [19].

Ovariectomized animals received estrogen treatment restored the thickness of the atrophied lingual mucosa [20, 21] and vaginal mucosa [22, 23]. Although hormonal replacement therapy improves oral mucosa alteration, long term hormonal treatment in menopause allows several hazards including thromboembolism and reproductive tissue cancer [21, 24], which obligate to explore a novel strategy for handling [25]. Herbal therapy including Remifemin was found to exert comparable effects to hormones [21], nevertheless, uninterrupted intake of herbal therapies is mandatory. Therefore, the goal now is to find a substitute therapy with long-lasting curing effect i.e. cell-based therapy [26, 27].

The utilization of stem cell-based therapies has sparked interest in the medical management of damaged tissues [27]. Transplantation of these cells to a specific tissue promotes regeneration via their self-renewal capacity and differentiation potentiality [28]. Mesenchymal stem cells (MSCs) of different sources including adipose tissue [29], dental pulp [30], umbilical cord [11], gingiva [31] and bone marrow [10, 32–34] were utilized in treating various tissue injuries including damage in parotid gland induced by irradiation [29, 31], by diabetes mellites [30], and by menopause [11, 12]. In addition, in treating submandibular gland injuries induced by cadmium chloride [32], tongue damage in diabetic rats [35], in osteoporosis treatment [36] and in the submandibular gland [10] of ovariectomized rats.

Bone marrow mesenchymal stem cells (BM-MSCs) show high regenerative potential in the maxillofacial region [10, 32–34, 37, 38]; however, their potential role in ameliorating the histological alterations in the lingual mucosa of menopausal rats is still unknown. Therefore, the aim of this study is to shed light on the possible efficacy of transplanted BM-MSCs on the structural changes in the filiform and fungiform papillae of the ovariectomized rats. Our null hypothesis proposed that BM-MSC transplantation does not affect the prospective histopathological changes in the filiform and fungiform papillae.

## Materials & methods

### Ethical statement

Animal handling and research methodology were performed after the permission of the Research Ethics Committee of Faculty of Dentistry, Minia University (Decision No. 912, Ethical Code: RHDIRB2017122001), and followed the national guidelines, the Guide to the Care and use of laboratory animals and the guidelines of the ARRIVE [39, 40].

### Preparation of BM-MSCs

Rats’ tibias and femora bone marrow were dissected from 3-week-old rats with full culture media DMEM-low glucose (1.0 g/L glucose, Lonza) added to 2 mM L-glutamine, 1% penicillin/streptomycin (P/S, 10.000IU/ml/10.000 µg/ml, Lonza), 10% fetal bovine serum (FBS, HyClone). After centrifuging, flushing bone marrow, the cell aggregates were suspended once more and moved to a T − 25 flask then kept in an incubator of CO_2_. The culture media was replaced two times a week, while the non-adherent cells were eliminated by PBS rinsing. Trypsin in 1 mM EDTA was used to separate the adhering cells after 80–90% confluence, then they were re-immersed at 0.5 × 10^6^ cells/ml in culture media for transplanting. To monitor the cultivated cells, a phase contrast inverted microscope was used. (Nikon TSM).

### Characterization of BM-MSCs by flow cytometry

**Fig. 1:**
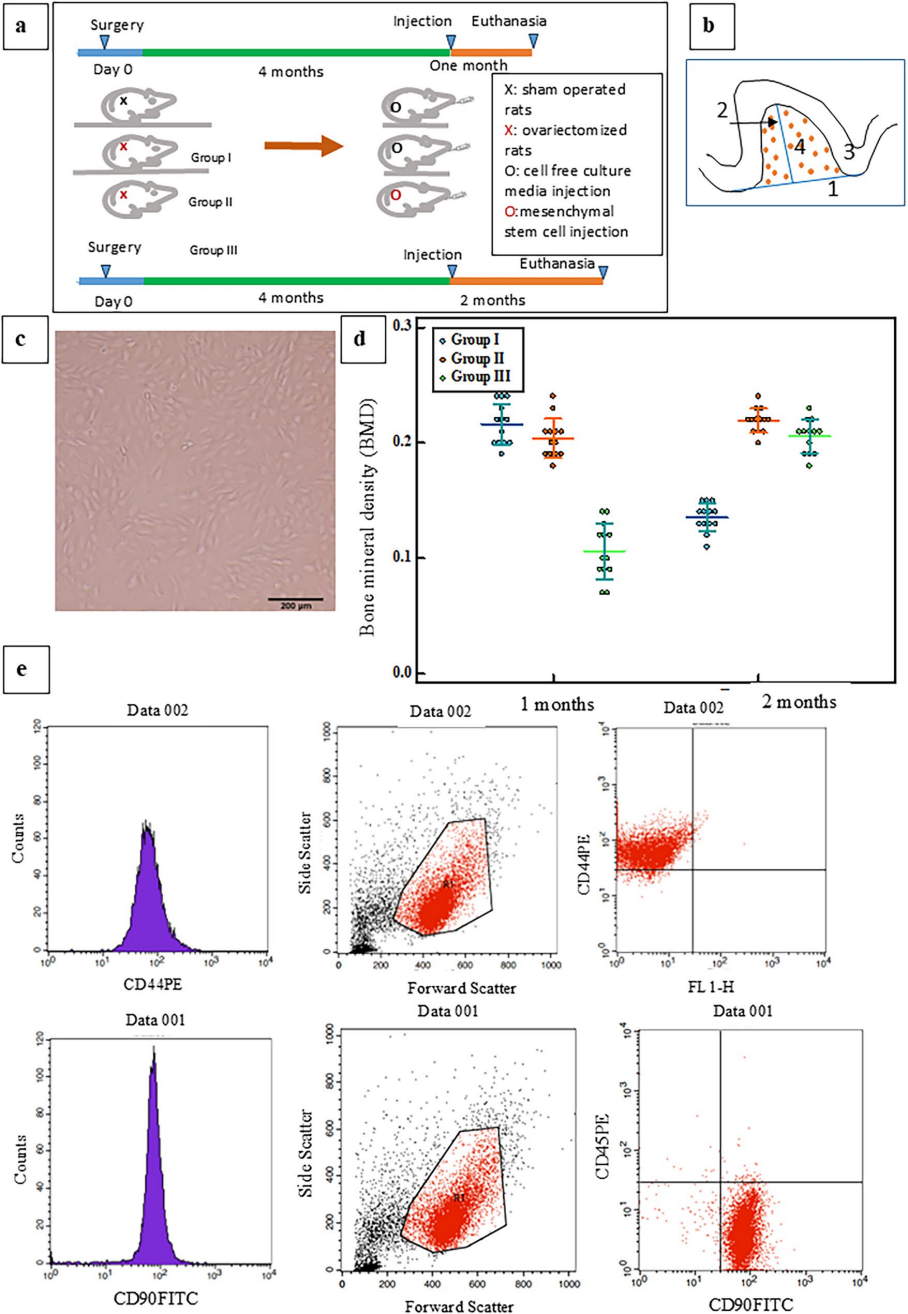
Experimental groups´ assessment of bone mineral density and characterization of the isolated stem cells. **a** Schematic diagram illustrating the experimental groups and the timeline of the experiment. **b** measurements of the area and height of the connective tissue papilla. The lowest points of the epithelial rete pegs are connected by line 1, thereafter line 2 is drawn from the top of the connective tissue papilla perpendicular to line 1. Line 2 represents the height of the connective tissue papilla. The traced area above line 1 and below the surface epithelium (3) represents the area of the connective tissue papilla (4). **c** is a photomicrograph of the spindle-shaped mesenchymal stem cells (x100). **d** is a graph of the comparison between the three investigated groups in relation to the Bone mineral density (BMD) of the femur. The BMD of the ovariectomized rats is significantly lower from the sham operated rats, and from that of the ovariectomized rats treated with BM-MSCs at one month and two months post-treatment respectively. Group I: sham operated rats, Group II: ovariectomized rats, Group III: ovariectomized rats treated with BM-MSCs. **e** Forward scatter represents the size of cultured cells, the side scatter for granularity. Unstained cells were utilized instead of isotype controls and compensation was performed by FITC and PE. Immunophenotyping of P3, mesenchymal stem cells, revealed that 96.35% of the cultured cells express CD44, 97.94% express CD90 mesenchymal stem cell markers, and 0.22% express hematopoietic cell marker. (Group I: sham operated rats, Group II: ovariectomized rats, Group III: ovariectomized rats treated with BM-MSC)

The stickiness and fusiform shape of MSCs in culture were assessed morphometrically using a phase-contrast light microscope (Olympus^®^, Tokyo, Japan) at a magnification of x100 (Fig. 1c). To further confirm MSC identity, fluorescent-labeled monoclonal antibodies of MSC surface markers (CD90-FITC, CD44-PE), and hematopoietic (CD45-PE) surface markers (Abcam, Cambridge, UK) were utilized [41, 42].

### Experimental design

In this investigation, 78 female albino rats aged 6–8 weeks and weighing 200–220 gm were used. The rats were obtained from the experimental animal house, medical physiology department at Alexandria university. Rats had unlimited access to water and a soy-free diet. The humidity and room temperature were preserved at 60% and 23 °C, respectively with stabilized light cycle at 12 h. The required number in each group was determined after a priori power analysis using G Power 3.1 9.2 software (University of Dusseldorf, Dusseldorf, Germany). Data obtained from previous literature [22] were used to calculate the effect size of (Cohen’s d ≈ 2.3) based on the Mean area percent of collagen fiber content. Assuming two-tailed alpha level of 0.05 and 80% statistical power, This yielded a minimum sample of 72 samples (24 per group). The final sample size increased to 78 (26 rats per group) to account for 10% dropout and allows subgroup analysis at multiple time points (day 30, and day 60), with 13 animals euthanized per group at each time (Fig. 1a).

### Surgical procedure

The rats received intraperitoneal injection of ketamine hydrochloride at a dose of 50 mg/kg and 5 mg/kg xylazine as an anesthetic solution prior to the operation [43]. Both ovaries were exposed via making an incision at the lower abdomen’s midline. The ovaries in the sham group (group I) were left intact without being excised. For rats of groups II & III, the uterine horns were ligated, and the ovaries were removed bilaterally followed by closure of the abdominal wound. Each rat was intramuscularly injected with 0.1 ml Penicillin G procaine (300,000-unit/ml, Phoenix Pharmaceutical Inc., St. Joseph, MO) as a preventive therapy for three days [44]. 25 mg/kg metamizole sodium analgesic was administered post-surgically two times a day for two days [45].

Rats were randomly assigned to three groups, (*n* = 26) for each, by utilizing random number generator. Group I included the sham operated rat. Group II included rats with experimentally induced ovariectomy [10]. Group III included rats with experimentally induced ovariectomy which received BM-MSC injection. The animals in each group were further subdivided equally into 2 subgroups (a & b, 13 rats in each subgroup) according to the date of euthanasia. The outcome assessor was blinded to the group assignment throughout the assessment. None of the animals were excluded.

Four months after surgery [20], groups I and II were dosed with single, localized injection of 0.25 ml cell-free media at the ventral tongue surface. Group III received single, local injection of 0.25 ml of culture media carrying 0.5 × 10^6^ of BM-MSCs [28] at the right side of the ventral surface of the tongue, midway between the lateral border and the midline of the tongue. Prior to injection, all rats were anesthetized via intraperitoneal injection of ketamine hydrochloride at a dose of 50 mg/kg and 5 mg/kg xylazine mixture [43].

Animals of groups Ia, IIa, IIIa were euthanized after one month post treatment [22], and two months post treatment [46] for groups Ib, IIb, IIIb, by intraperitoneal injection of sodium pentobarbital 40 mg/kg [34] followed by cervical dislocation of the heads.

### Dual-energy X-ray absorptiometry (DEXA) analysis

The bone mineral density (BMD) of the rat femur was assessed (*n* = 13 per subgroup) utilizing the DEXA scan (MEDIX DR, 2D Fan Beam DEXA, The National Research Center, Medical Service Unit, Dokki, Cairo, Egypt). The dissected femur was placed on the X-ray table, and the sample was scanned to measure the BMD. A fine X-ray beam of a minimum dose penetrate the sample, as the scanner arm move above the samples. A detector in the scanner arm assesses the amount of the X-ray that penetrated the samples [47].

### Histopathological examination

At each euthanasia date, the tongues of 8 rats/subgroup [10] were dissected. The anterior two thirds of the tongues were sectioned anterior to the circumvallate papillae in each subgroup. Samples were immediately placed in formaldehyde (10%) for 48 h. The tongue samples were sectioned into two halves along the midline, thereafter, dehydrated in (70%, 95%, 99.5%) alcohol, 3 baths for each concentration and 30 min per change. Thereafter, the specimens were cleared in (5–6 baths) of xylene, 5 min each bath. Then, the tissue samples were infiltrated by 5–6 changes of wax, 20 min/change, and finally embedded in paraffin. Sagittal sections of 5 μm thickness were obtained. The tissue sections were mounted on glass slides (StarFrost, Kitteglass, Germany) followed by staining by H&E (Eosin stain, C.I.45380, Cambrian chemicals; Hematoxylin according to Harris solution, C75.I. 290, Cambrian Chemicals) for histological examination [48, 49] and Masson trichrome utilizing the Masson Trichrome Staining Kit (Light Green) Masson 1929 (RRSK21-100, Atom Scientific, Machester, UK) to evaluate the collagen distribution [10, 50]. Stained tissue sections were assessed using a light microscope fixed with a digital camera (DM LB100T, LEICA; digital camera- DFC295- LEICA Microsystems, Germany). The photomicrographs were obtained at a magnification of x200 and x400.

### Scanning electron microscopic (SEM) examination

After euthanasia, 5 tongue samples [51] from each subgroup were rinsed in 0.1 M. After 1.5–2 h of fixing in 2.5% glutaraldehyde in phosphate buffer, the specimens were transferred to a post-fixative (2% osmium in phosphate buffer) for a further 2 h. Ethanol was serially diluted for 10 min at each concentration in order to dehydrate the samples. The critical point dryer substituted CO_2_ for ethanol at high pressure (Audosamdri-815, Tousimis; Rockville, Maryland, USA). Gold was applied to the samples using an extra cold sputter coater (SPI-module) [28]. Tongue samples were later scanned by JSM-5300 scanning electron microscope.

### Histomorphometric analysis

Image J software (NIH, version 1.53e v) was used to analyze the images of specimens. Five different images from each animal sample (8 rats per subgroup, *n* = 8 rats) were selected, consistently from the anterior 2/3 of the tongue and specifically from the filiform papillae, from H&E and Masson trichrome stained tissues. Magnification of x400 to measure the height, and area of the connective tissue papillae, collagen area% of the connective tissue papillae, and x 200 to measure the keratin thickness of the inferior lingual epithelium were utilized. The mean values were averaged per animal. Calibration was set utilizing a stage micrometer (1 pixel = 0.1945 μm, equivalent to 5.14 pixels/µm) and was consistent for the scale bars confirming accuracy among images. All measurements were reported in micrometers (µm). The assessor was blinded to group assignment.

The region of interest of the connective tissue papilla was manually defined and the height and area of the connective tissue papillae were measured within the traced area (Fig. 1b). For the height of the connective tissue papilla, the lowest locations of the two nearby epithelial rete pegs were defined by a line, thereafter a line was drawn from the top of the connective tissue papilla (at the junction between the epithelium and the papillary connective tissue) perpendicular to the preceding line which represent the connective tissue papilla height [52]. To analyze the connective tissue papillae area, the space occupied by the connective tissue papilla was identified and measured. The free hand selection tool was utilized to trace the junction between the surface epithelium and the underlying connective tissue, including the indentation into the epithelium was selected to measure the connective tissue papillae area [52].

In addition, the collagen area% of the connective tissue papillae was analyzed. The Color Threshold tool was utilized. At the beginning, the calibration of the software was done for converting the pixels to micrometer measurement units. Thereafter, the images were converted to grey delineated images followed by masking the selected area by red binary color. The threshold range was (140–220). The region of interest was manually delineated using the free hand selection tool to trace the connective tissue of the largest papillae. The mean value for each sample was calculated [53].

The keratin thickness of the lingual epithelium was assessed in the inferior lingual surface only, as the dorsal surface of the tongue is covered by lingual papilla projections which protrude above tongue dorsal surface. Furthermore, the keratin thickness covering the keratinized papilla is not uniform, being thicker at the tip than at the lateral borders. To measure the thickness of the keratin layer of the lingual epithelium of the inferior surface of the tongue, twenty points were selected on a photomicrograph from the tongue tissue section, utilizing magnification x 200. The points were evenly spaced, and points were selected utilizing grid (line type). The mean thickness was then calculated [20].

### Statistical analysis

Statistical analyses for height, area of connective tissue papillae, keratin thickness f the inferior lingual epithelium and collagen area% were performed utilizing the Statistical Package for the Social Sciences version 27.0 (Armonk, NY: IBM Corp). Shapiro-Wilk test was utilized to assess the normality of the measured variables. The data were normally distributed, therefore, parametric tests were utilized. Numerical variables were represented as range (minimum and maximum), mean±standard deviation, standard error of the mean, median and interquartile range. To compare between the three groups, one-way analysis of variance (ANOVA) followed by Bonferroni-adjusted post hoc test were utilized when the assumption of homogeneity was satisfied. The homogeneity was assessed by Leven´s test and when violated, Welch´s ANOVA was employed followed by Games-howell post hoc tests for pairwise comparisons.tpo compare between one month and two month´s measurements, independent sample student t-test was utilized. Effect size for between group comparisons was quantified utilizing η² with 95% confidence intervals (CI). All statistical tests were two-tailed and the exact *p*-value was reported. *P*-value < 0.05 denotes significant results.

## Results

### Characterization of BM-MSCs

Immunophenotyping of the isolated fusiform-shaped BM cells revealed that 96.35% of the cultured cells express CD44, 97.94% express CD90 mesenchymal stem cell markers, and 0.22% express CD45 (Fig. 1c, e).

### Low BMD in the ovariectomized rats

To confirm estrogen deficiency in the ovariectomized rat model (13 rats per subgroup, *n* = 13 rats), the bone mineral density (BMD) was assessed. The ovariectomized rats depicted low BMD at one month post injection (0.135 ± 0.012, 95% CI of the mean: 0.127–0.142) at one month post injection and (0.107 ± 0.025, 95%CI of the mean 0.092–0.122) at two month post injection, compared to the sham operated rats at one month (0.216 ± 0.017, 95%CI of the mean 0.206–0.227) and two months (0.218 ± 0.011, 95%CI of the mean 0.211–0.224) post-treatment with (MD = 0.081, 95%CI: 0.066–0.096, *P* = < 0.001) at one month and (MD = 0.111, 95%CI: 0.092–0.130, *P* = < 0.001) at two month post treatment. The BMD in the ovariectomized rats treated with BM-MSCs was significantly higher than that of the ovariectomized rats (*p* < 0.001) at one month (0.203 ± 0.016, 95%CI of the mean 0.193–0.203) and two months (0.205 ± 0.014, 95%CI of the mean 0.196–0.213) post treatment with (MD = 0.69, 95%CI: 0.054–0.083, *p* = < 0.001) at one month and (MD = 0.098, 95%CI: 0.078–0.118, *p* = < 0.001) at two month post treatment (Fig. 1d, Table 1).

**Table 1:** Comparison between the three studied groups according to Bone mineral density (BMD)

Bone mineral density (BMD)	Sham (*n* = 13)	OVX (*n* = 13)	OVX + MSC (*n* = 13)	Test of Sig. (df), *p*	Post Hoc Test			Effect Size
					Sham vs. OVX MD (95% C.I), *p*	Sham vs. OVX+ MSC MD (95% C.I), *p*	OVX vs. OVX+ MSC MD (95% C.I), *p*	η² (95% C.I)
One month								
Min. – Max.	0.194–0.244	0.105–0.150	0.184–0.235	F_(2,36)_ = 102.346^*^, *p* < 0.001^*^	0.081 (0.066–0.096), *p* = < 0.001^*^	0.013 (-0.002–0.028), *p* = 0.135	0.069 (0.054–0.083), *p* = < 0.001^*^	0.850 (0.735–0.893)
Mean ± SD. (SE)	0.216 ± 0.017 (0.005)	0.135^#^ ± 0.012(0.003)	0.203^$^ ± 0.016(0.005)					
95% C.I. of mean	0.206–0.227	0.127–0.142	0.193–0.213					
Median (IQR)	0.216 (0.201–0.229)	0.139 (0.129–0.142)	0.203 (0.190–0.210)					
Two month								
Min. – Max.	0.197–0.240	0.066–0.143	0.180–0.230	^#^F_(2,22.24)_ = 107.955^*^, *p* < 0.001^*^	0.111 (0.092–0.130), *p* = < 0.001^*^	0.013 (0.001–0.026), *p* = 0.036^*^	0.098 (0.078–0.118), *p* = < 0.001^*^	0.896 (0.814–0.926)
Mean ± SD. (SE)	0.218 ± 0.011 (0.003)	0.107^#@^±0.025(0.007)	0.205^$^ ± 0.014(0.004)					
95% C.I. of mean	0.211–0.224	0.092–0.122	0.196–0.213					
Median (IQR)	0.217 (0.213–0.221)	0.100 (0.091–0.124)	0.207 (0.194–0.210)					
MD (95% C.I)	0.002(-0.010 − 0.014)	0.028 (0.012–0.044)	0.001(-0.011–0.014)					
t, p_0_	t = 0.311,p_0_ = 0.759	t = 3.659^*^,p_0_ = 0.002^*^	t = 0.217,p_0_ = 0.830					

*n* = 13 rats

*IQR* Inter quartile range, *SD* Standard deviation, *SE* Standard error of mean, *MD* Mean difference (Higher – Lower), *CI* Confidence interval, *df* degree of freedom

t: independent samples student t-test

F: F for One way ANOVA test, Pairwise comparison bet. each 2 groups was done using Post Hoc Test (adjusted Bonferroni)

p: *p* value for comparing between the three studied groups

p0: *p* value for student t-test for comparing between One month and Two month in each group

#F: Welch ANOVA test, Pairwise comparison bet. each 2 groups was done using Post Hoc Test (Games-Howell)

*: Statistically significant at *p* ≤ 0.05

#: Significant with Sham, $: Significant with OVX, @: Significant between One month and Two month

### Abnormal lingual mucosa and surface structure of the ovariectomized rat tongue and amelioration of the alterations after one month of BM-MSC injection

**Fig. 2:**
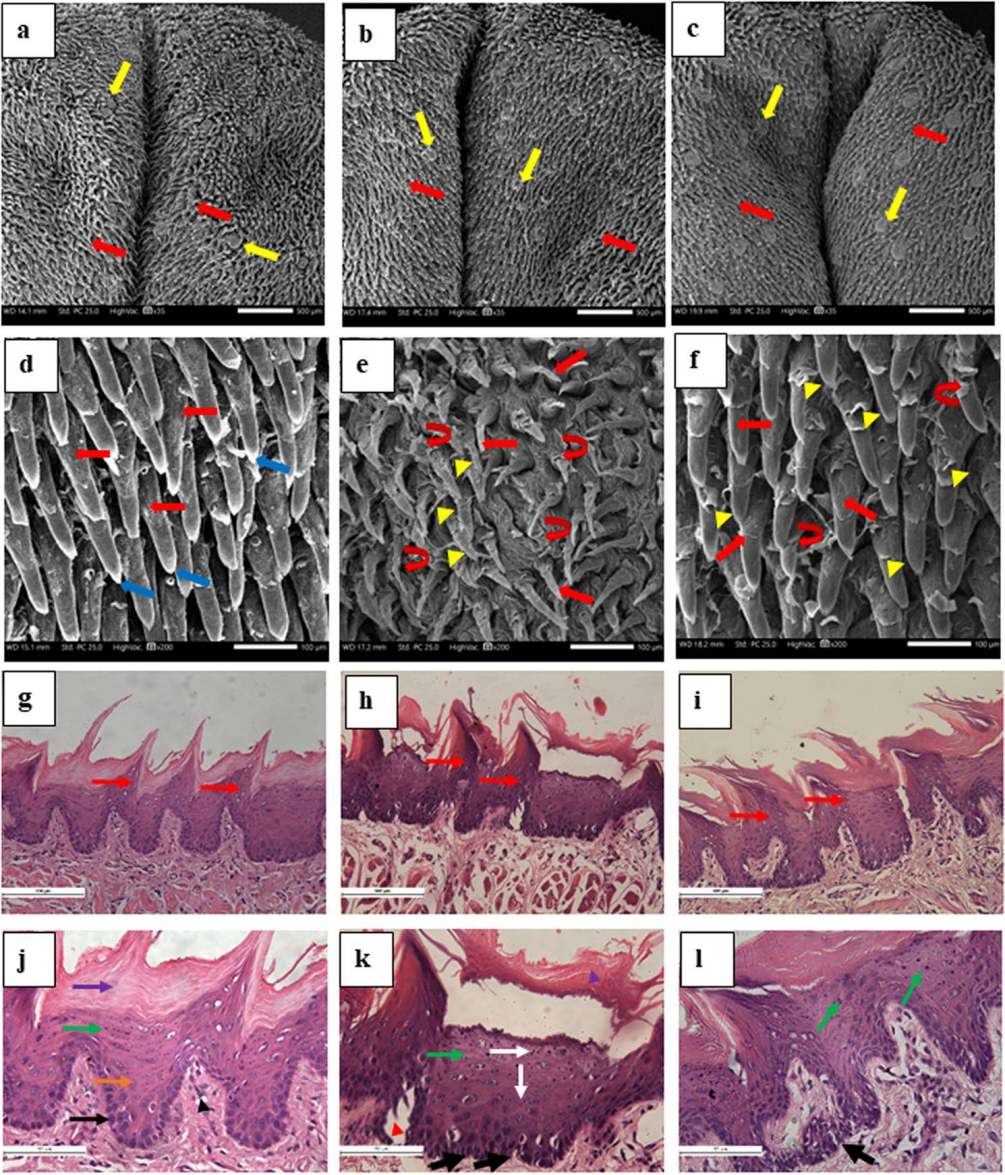
Promoted recovery of filiform papilla normal architecture in stem cell-treated rats, one month post treatment. Scanning electron micrographs of dorsal surface of rats’ tongue, one month post treatment, show (**a**, **d**) for group I; (**b**, **e**) for group II; (**c**, **f**) for group III. **a**, **b**, **c**: numerous filiform papillae (red arrow) with scattered fungiform papillae in-between (yellow arrows). **d** parallel rows of filiform papillae with long finger-like processes (red arrow), keratinized pointed or rounded end papillae (blue arrow). **e** irregularly organized filiform papillae with thinning along their length and eroded ends (red arrows), distorted filiform papillae (curved red arrow) with detached and desquamated threads of keratin (yellow arrowhead). **f** almost uniform filiform papillary orientation (red arrow), few remaining signs of degeneration (curved red arrow) with surface cracks and keratin flakes (yellow arrowhead). [SEM, (a-c) x35, (D-F) x200]. **g**-**l** are photomicrographs of filiform papillae. **g** group I with conical shaped filiform papillae (red arrow), (**h**) group II with irregular pattern of filiform papillae (red arrow) and (**i**) group III with near regain of normal filiform papillae (red arrow). **j**-**l** are higher magnification photomicrographs of (**g**-**i**) respectively. **j** neatly arranged basal cells (black arrow), polyhedral prickle cell layer (orange arrow), flattened granular layer with keratohyalin granules (green arrow), thick intact keratinous layer (purple arrow), interlacing collagen fibers and scattered fibroblasts (black arrowhead), (**k**) irregular basal cells (black arrows), area of detachment (red arrowhead), some granular cells with no definite boundaries and degeneration (green arrow), scattered keratohyalin granules in prickle and granular cells (white arrow) and thin, detached keratin threads (purple arrowhead), (**l**) areas of irregular basal cells with fuzzy appearance of basement membrane (black arrow) and keratohyalin granules confined to the granular cells (green arrow). [H&E, (**g**-**i**) x200, (**j**-**l**) x400]

The dorsal lingual epithelium of the three studied groups was loaded mainly with numerous filiform papillae and scattered fungiform papillae in-between (Fig. 2a-c). Group Ia revealed normal filiform papilla architecture. The papillae appeared tall, conical shaped, directed anteroposterior and assuming organized parallel rows of same direction. They had wide base and uniformly keratinized pointed or rounded ends with clear papillary margins and smooth surface (Fig. 2a, d). The epithelium displayed uniformly arranged strata with long and well-developed epithelial ridges and connective tissue papillae. The underlying lamina propria demonstrated normal appearance of collagen fibers, muscle bundles and blood vessels (Figs. 2g, 6a). Higher magnification showed neatly arranged basal cells. The spinous cells appeared polyhedral, while the granular cells were flattened and contained numerous basophilic keratohyalin granules. An overlying thick and intact keratinous layer was also spotted. Numerous interlacing collagen fibers and deeply scattered basophilic fibroblasts were detected in the underlying connective tissue (Fig. 2j). The filiform papillae in group IIa, showed noticeable thinning along their length with eroded ends. They were distorted and irregularly organized showing dissimilarity in their orientation and inclination. Some papillae revealed severe loss of their normal conical configuration. The papillary surface appeared rough with cracks and detached, desquamated threads of keratin (Fig. 2b, e). The papillae depicted irregular histology with detached overlying keratin. The epithelial ridges appeared short and few with areas of flattening. However, the lamina propria consisted of thin loosely arranged collagen fibers with an apparent decrease in the height of the connective tissue papillae. The underlying collagen and muscle bundles lost their compactness showing signs of atrophy and degeneration (Figs. 2h, 6b). The basal cells lacked uniform arrangement on the basement membrane and displayed some areas of detachment. Some granular cells showed no definite cell boundaries and degeneration in some areas. The keratohyalin granules were scattered within prickle and granular cell layers. Detached and irregular interlacing threads of keratin were clearly identified (Fig. 2k). In group IIIa, the filiform papilla displayed almost uniform papillary orientation, exhibiting conical pattern and rounded keratinized tips. However, they appeared rough with surface cracks and keratin flakes (Fig. 2c, f). The papillae started to revert to their typical structure, with focal areas of detached keratin were still noticed. Epithelial ridges exhibited nearly normal configuration alternating with regular connective tissue papillae. The lamina propria revealed few areas of degeneration among thin interlacing collagen fibers and muscle bundles (Figs. 2i, 6c). Altered basal cells architecture characterized by lack of uniform arrangement was still noticed in some areas and the keratohyalin granules were confined to the normally configured granular cell layer (Fig. 2l).

**Fig. 3:**
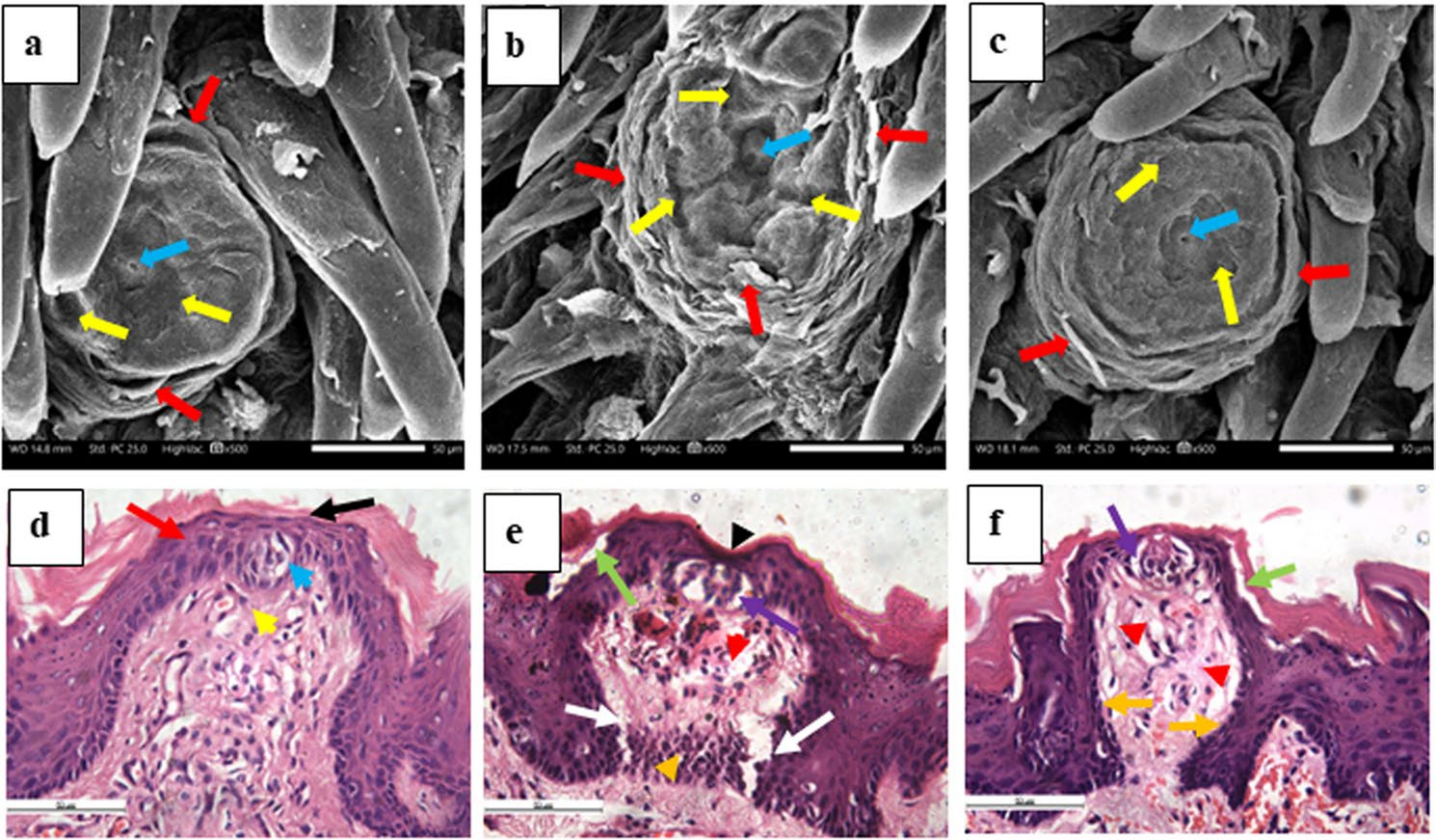
Promoted recovery of fungiform papilla normal structure in stem cell-treated rats, one month post treatment. **a**-**c** are scanning electron micrographs of dorsal surface of rats’ tongue, one month post treatment. Group I (a) dome-shaped fungiform papillae with elevated margins and thin keratin flakes (red arrows), shallow indentations on the surface (yellow arrows), well-defined taste hole (blue arrow). Group II (**b**) ill-defined raised boundaries of fungiform papillae and torn keratin scales (red arrows), numerous surface indentations (yellow arrows) depressed eccentric gustatory pore (blue arrow). Group III (**c**) sharp high up edges of fungiform papilla with slightly irregular keratinized surface (red arrows), few surface indentations (yellow arrows), distinct taste bud with patent gustatory pore (blue arrow). [SEM, (**a**-**c**) x500]. **d**-**f** are photomicrographs of fungiform papillae. Group I (**d**) mushroom-like fungiform papilla with normal keratinized stratified squamous epithelium (red arrow), thin keratinous layer (black arrow), onion-shaped taste bud (blue arrowhead), interlacing collagen fibers and scattered basophilic fibroblasts (yellow arrowhead). Group II (**e**) disfigured fungiform papilla with depressed central core (black arrowhead), area of detached keratin (green arrow), irregular cuboidal or flattened basal cells with increased cellularity and indistinct basement membrane (white arrow), vacuolated taste bud (purple arrow), sparse collagen fibers with areas of degeneration (red arrowhead), inflammatory cell infiltrate (orange arrowhead). Group III (**f**) fungiform papilla with areas of detached keratin (green arrow), flattened basal cells (orange arrow), minor degeneration of the taste bud peripherally (purple arrow), and some areas of matrix distortion (red arrowhead). [H&E, (**d**-**f**) x400]

Fungiform papillae of group Ia depicted dome shaped fungiform papillae and were randomly scattered between the filiform ones. The papillae exhibited elevated rounded margins with thin keratin flakes and shallow indentations on the surface. Regular well-defined taste hole was recognized as a centralized depression within the papilla (Fig. 3a). The papillae exhibited normal keratinized stratified squamous epithelium architecture. The surface epithelium revealed columnar basal cells of deeply stained basophilic nuclei, polyhedral prickle cells, flattened granular cells and thin overlying keratinous layer. Onion shaped solitary taste bud was detected within the papillary dorsal epithelium. The connective tissue showed abundant interconnected collagen bundles, basophilic fibroblasts and numerous blood vessels (Fig. 3d). In group IIa, the fungiform papillae showed irregular, ill-defined raised boundaries of microridges and torn keratin scales on the papillary epithelial covering with numerous indentations. The taste bud appeared to be depressed while the gustatory pore seemed to be eccentric (Fig. 3b). The papilla and the taste bud exhibited disfigurement. The overlying epithelium and keratin appeared thin with depressed central core and areas of detached keratin. On the lateral walls of the papilla, the basal cells showed some irregularities in their arrangement and shape and some of them appeared cuboidal or flattened with indistinct basement membrane. The taste bud was vacuolated showing signs of degeneration. The connective tissue core displayed sparse collagen fibers, inflammatory cell infiltrate and some areas of degeneration (Fig. 3e). In group IIIa, papillae revealed clear sharp high up edges along with slightly irregular keratinized surface and few indentations. Well-defined taste bud with patent gustatory pore could be recognized in the center of the papillary surface (Fig. 3c). The regular structure of the fungiform papilla and bud was nearly restored. However, areas of detached keratin were still noticed. Flattening of basal cells was consistently observed on both papillary sides. Distinct barrel-shaped taste bud was visible at the papillary summit with minor degeneration peripherally. The papillary connective tissue revealed scattered, interlacing collagen bundles with some areas of matrix distortion (Fig. 3f).

### Promoted recovery of the regular lingual mucosa histology and surface structure of the dorsal surface of the tongue in the BM-MSC-treated ovariectomized rat after two months post-injection

**Fig. 4:**
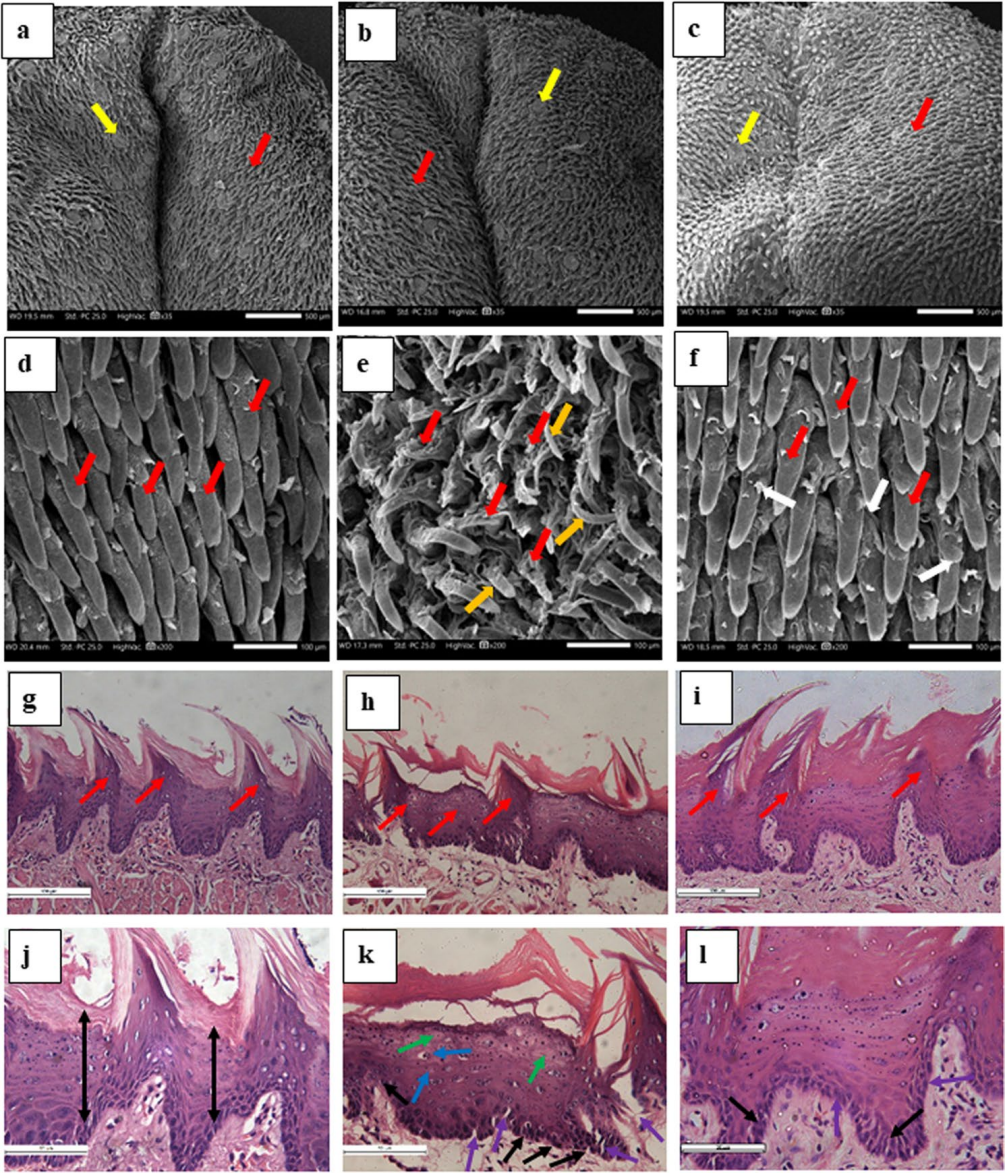
Stem cell treated rats are associated with regain of filiform papilla normal structure after two-months post injection. Scanning electron micrograph of dorsal surface of rats’ tongue, two months post treatment shows (**a**, **d**) for group I, (**b**, **e**) for group II and (**c**, **f**) for group III. **a**-**c** are micrographs of numerous filiform papillae (red arrow) with scattered fungiform papillae in-between (yellow arrow). **d** conical-shaped filiform papillae with regular direction and tapered keratinized tips (red arrow). **e** disfigured filiform papillae with atrophied and shredded keratin threads (red arrow), thin and bent papillae with loss of keratin (orange arrow). **f** shows tapered evenly directed filiform papillae with hornified endings (red arrow), minimal desquamated keratin fragments (white arrow). [SEM, (**a**-**c**) x35, (**d**-**f**) x200]. **g**-**l** are photomicrographs of filiform papillae. **g** group I with conical shaped filiform papillae (red arrow), (**h**) group II with disappearance of normal filiform papillae with papillary detachment (red arrow) and (**i**) group III with normal filiform papillary pattern and preserved keratin tips over most papillae (red arrow). **j**-**l** are higher magnification photomicrographs of (**g**-**i**) respectively. **j** shows normal strata of keratinized stratified squamous epithelium (black double arrow). **k** shows cuboidal and crowded basal cells with disrupted architecture (black arrows), areas of basement membrane separation (purple arrow), enlarged granular and spinous cells with pyknotic or degenerated nuclei (green arrow), numerous keratohyalin granules throughout the epithelial thickness (blue arrow). **l** shows some areas of disturbed uniformity of basal cells (black arrows), clearly outlined basement membrane (purple arrow), and abundant keratohyalin granules within the granular cells (black stars). [H&E, (**g**-**i**) x200, (**j**-**l**) x400]

Group Ib revealed a filiform papilla appearance similar to that of group Ia (Fig. 4a, d) and showed normal histology (Figs. 4g, j, 6d). In group IIb, most of filiform papillae were massively disfigured and haphazardly arranged. Some papillae seemed rough and atrophied with shredded keratin threads. Others were evidently thin and bent, with loss of uniform overlying keratin (Fig. 4b, e). The papillae depicted disappearance of normal papillary form coupled with keratin detachment. The covering epithelium lacked the known criteria of keratinized squamous epithelium with torn and separated keratin threads. Some epithelial ridges appeared ill-defined. The reticular layer presented loose and deteriorated collagen fibers with signs of atrophy of the muscle bundles. Wide vacant areas were spotted denoting complete degeneration with apparent loss of some connective tissue papillae (Figs. 4h, 6e). Some basal cells appeared cuboidal and crowded with disrupted architecture, together with areas of basement membrane separation. Granular and some spinous cells seemed to be enlarged, with pyknotic or degenerated nuclei. Except at the basal layer, numerous dispersed keratohyalin granules were identified throughout the epithelial thickness (Fig. 4k). In group IIIb, filiform papillae depicted normal papillary appearance with long, tapered, evenly directed orientation. They exhibited nearly smooth surfaces and blunt cornified endings (Fig. 4c, f). The papillae revealed restored normal papillary configuration. Epithelial strata appeared almost normal with preserved keratin tips over most of the papillae accompanied by well-developed epithelial ridges. Fine interlacing collagen fibers were clearly evident in the underlying lamina propria with restoration of connective tissue papillae (Figs. 4i, 6f) with some areas of disturbed uniformity of basal cells (Fig. 4l).

**Fig. 5:**
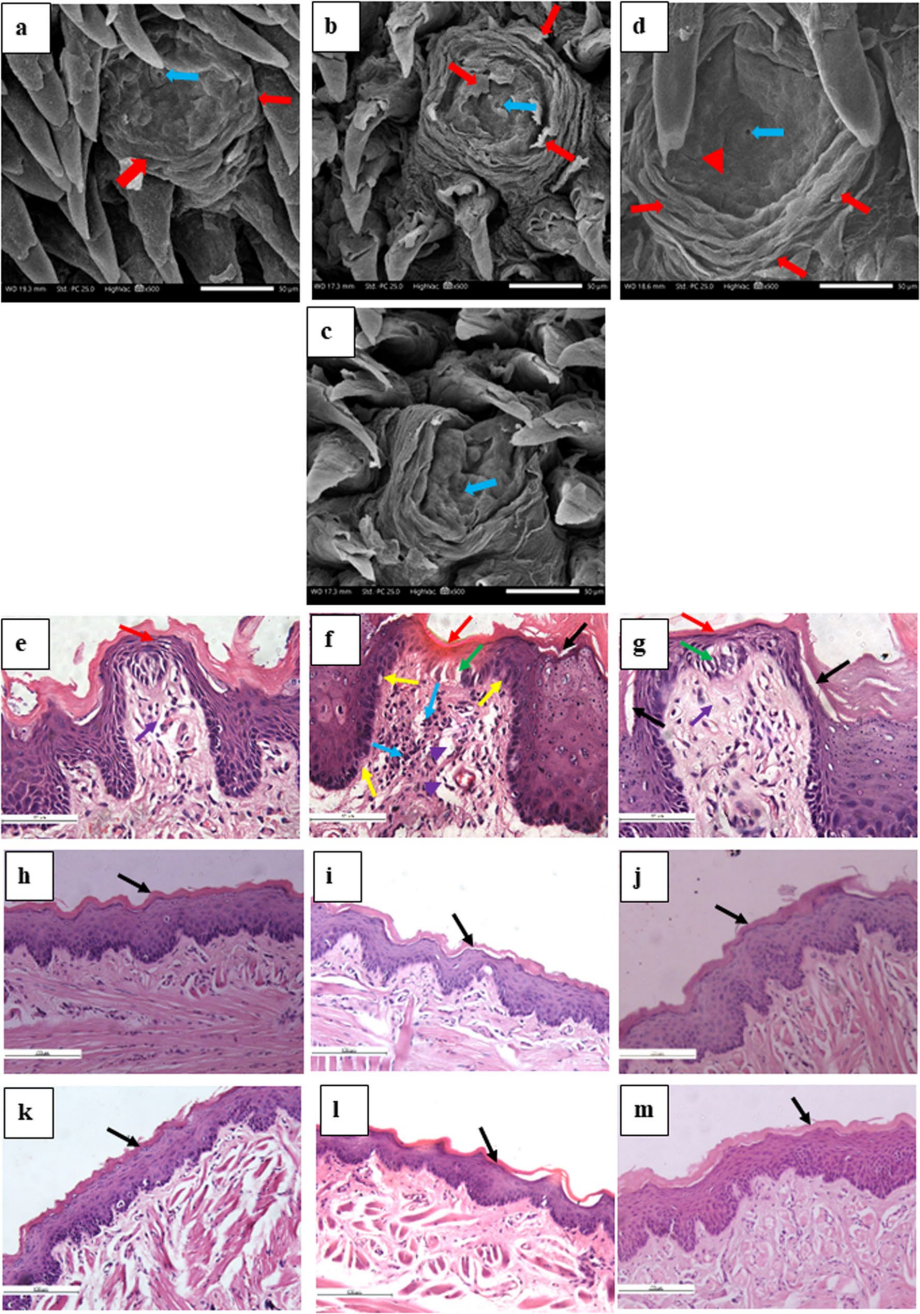
Stem cell treated rats are associated with restoration of normal fungiform papilla architecture two-months post injection and keratin thickness of inferior lingual epithelium. **a**-**d** are scanning electron micrographs of dorsal surface of rats’ tongue, two months post treatment (**a**) represents group I, (**b**, **c**) represent group II, and (**d**) represents group III. **a** a mushroom-shaped fungiform papilla with well-identified outlines and keratinized surface (red arrows) and distinct taste hole (blue arrow). **b** disfigured fungiform papilla with misshapen elevated borders and shredded keratin (red arrows) and undefined taste pore (blue arrow). **c** disfigured fungiform papilla (red arrow), atrophied taste buds and obliterated taste pore (blue arrow). **d** uniformly shaped fungiform papilla with slightly irregular and raised epithelial microridges (red arrows), smooth papillary surface (red arrowhead) and unblocked taste pore (blue arrow) [SEM, (**a**-**d**) x500]. **e**-**g**, (**h**-**m**) are photomicrographs of fungiform papillae and lingual mucosa of the inferior surface of the tongue. Group I (**e**) A mushroom-shaped fungiform papillae with taste bud (red arrow) and well-developed connective tissue core (purple arrow). Group II (**f**) A deformed fungiform papilla with atrophied epithelial central depression and thin keratin (red arrow), separated keratin filaments (black arrow), disturbed basal cells and blurred appearance of the basement membrane (yellow arrows), degenerated and vacuolated taste bud (green arrow) loose fibers of collagen and degenerated areas (purple arrowhead), and inflammatory cell infiltrate (blue arrows). Group III (**g**) uniform fungiform papillary pattern with keratin thinning while reaching the papillary surface (red arrow), areas of keratin detachment (black arrow), well-structured taste bud (green arrow) and dense organized collagen fibers (purple arrow). Group I (**h**, **k**) inferior lingual epithelium with regular thickness of the keratin layer at one month (**h**) and two month (**k**) post treatment (black arrow). Group II (**i**, **l**) apparent thinning of the keratin layer of the inferior lingual surface at one month (**i**), and two months (**l**) post treatment (black arrow). Group III (**j**), (**m**) promoted recovery of keratin thickness of the inferior lingual epithelium at one month (**j**) and two months (**m**) post-treatment (black arrow) [H&E, (**e**-**g**) x400, (**h**-**m**) x200]

The tongue of group Ib revealed fungiform papillae with mushroom shape appearance and clearly distinct taste hole (Fig. 5a). The papillae and the taste bud are normally structured with well-developed fibrous connective tissue core (Fig. 5e). In group IIb, the papillae were disfigured showing loss of their characteristic outline (Fig. 5b, c). Rough misshapen elevated borders giving wrinkled appearance with numerous shredded keratin were noticed on the surface (Fig. 5b). However, the taste buds appeared atrophied and deformed with blurred or obliterated gustatory pore (Fig. 5b, c). The fungiform papilla and taste bud were deformed. A widened area of atrophied epithelial central depression and thin overlying keratinous layer was spotted. The surrounding keratin filaments were shredded and separated. Disturbed architecture of basal cells was found on the peripheral walls of the papilla. The taste bud was degenerated with vacuolization while the lamina propria showed loose collagen fibers, focal areas of degeneration and inflammatory cell infiltrate (Fig. 5f). The tongue of group IIIb revealed uniform papillary pattern. Slightly irregular and raised epithelial microridges were detected surrounding nearly smooth papillary surface with unblocked taste bud pore (Fig. 5d). The papillae and the taste bud resumed their uniform structure. However, keratin detachment was still evident on lateral sides of the papilla. Dense organized collagen fibers of the underlying lamina propria were clearly seen (Fig. 5g).

### Decreased height, area and the collagen area percent of connective tissue papillae and keratin thickness of the inferior lingual epithelium in the ovariectomized rat tongue and their promoted restoration in the BM-MSC treated rats at one and two months post injection

Comparing the three investigated groups in relation to the height and area of the connective tissue papillae, collagen area percent of the connective tissue papillae and keratin thickness of the inferior lingual epithelium in one month groups post injection specimens, using one-way ANOVA and two-month post injection specimens using Welch´s ANOVA showed statistically significant difference (*p* < 0.001).

**Fig. 6:**
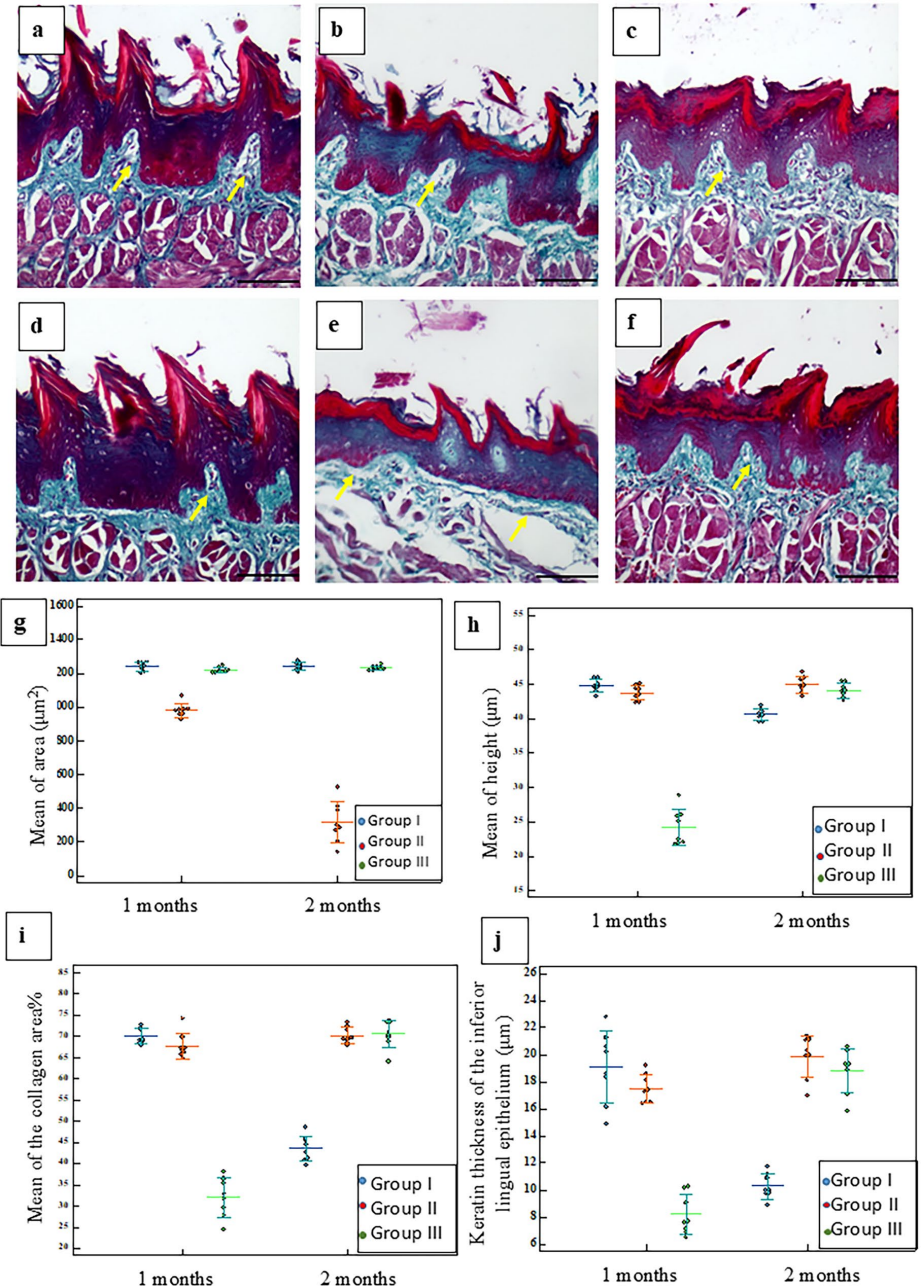
Stem cell-treated rats are associated with restoration of connective tissue papillae´ area, height and collagen area percent and keratin thickness of the inferior lingual epithelium. Photomicrographs of filiform papillae show (**a**, **b**, **c**) for groups I, II, III one-month post-treatment respectively and (**d**, **e**, **f**) for groups I, II, III two months post treatment respectively. **a** interlacing collagen fibers (yellow arrow). **b** degenerated collagen fibers (yellow arrow). **c** few areas of degeneration among the thin interlacing collagen fibers (yellow arrow). **d** well-developed fibrous connective tissue cores (yellow arrow). **e** loose collagen fibers with wide vacant areas (yellow arrow). **f** fine interlacing collagen fibers (yellow arrow) [Masson Trichrome, (**a**-**f**) x 400 magnification]. **g** is a graph showing a comparison between the investigated groups in relation to the area of connective tissue papillae in one- and two-months post treatment. **h** is a graph showing a comparison between the three studied groups according to the height of the connective tissue papillae in one- and two-months post treatment. **i** is a graph showing a comparison between the investigated groups in relation to the collagen area % of connective tissue papillae in one- and two-months post-treatment. **j** is a graph showing a comparison between the investigated groups in relation to the keratin thickness of the lingual epithelium of the inferior surface of the tongue. (Group I: sham operated rats, Group II: ovariectomized rats, Group III: ovariectomized rats treated with BM-MSCs)

For the height of the connective tissue papillae, the (mean ± SD, 95%CI of the mean) for group I was (44.82 ± 0.92, 44.06–45.59 μm) at one month, and (44.95 ± 1.18, 43.96–45.94 μm) two month post injection, for group II was (40.68 ± 0.87, 39.95–41.40 μm)at one month, (24.29 ± 2.66, 22.06–26.51 μm) at two month post injection, and for group III was (43.82 ± 1.06, 42.94–44.71) at one month and (44.16 ± 1.04, 43.29–45.03 μm) at two month post injection. Post hoc analysis utilizing adjusted Bonferroni test at one month post injection and Games-Howell test at two month post injection revealed that group II has decreased height compared to group I was (mean difference (MD) = 4.15 μm, 95% CI: 2.95–5.34, *p* = < 0.001) at one month, (MD = 20.67 μm, 95%CI:17.83–23.51, *p* = < 0.001) at two month post injection. In addition, group II has significantly decreased height compared to group III at one month (MD = 3.15 μm, 95% CI: 1.95–4.34, *p* = < 0.001), and at two-month (MD = 19.87 μm, 95%CI: 17.06–22.69, *p* = < 0.001) post injection. The effect size η² (95% C.I) at one month post injection was 0.798(0.569–0.865) and at two-month post injection was 0.970 (0.931–0.980) (Table 2, Fig. 6h).

**Table 2:** Comparison between the investigated groups in relation to the height of connective tissue papillae in in one- and two- months post treatment

Height (µm)	Sham (*n* = 8)	OVX (*n* = 8)	OVX + MSC (*n* = 8)	Test of Sig. (df), *p*	Post Hoc Test			Effect Size
					Sham vs. OVX MD (95% C.I), *p*	Sham vs. OVX+ MSC MD (95% C.I), *p*	OVX vs. OVX+ MSC MD (95% C.I), *p*	η² (95% C.I)
One month								
Min. – Max.	43.28–45.99	39.56–42.04	42.41–45.18	F_(2,21)_ = 41.507^*^, *p* < 0.001^*^	4.15 (2.95–5.34), *p* = < 0.001^*^	1.00 (-0.20–2.19), *p* = 0.144	3.15 (1.95–4.34), *p* = < 0.001^*^	0.798 (0.569–0.865)
Mean ± SD. (SE)	44.82 ± 0.92 (0.32)	40.68^#^ ± 0.87 (0.31)	43.82^$^ ± 1.06 (0.37)					
95% C.I. of mean	44.06–45.59	39.95–41.40	42.94–44.71					
Median (IQR)	44.82 (44.28–45.55)	40.66 (39.96–41.28)	43.94 (42.88–44.67)					
Two month								
Min. – Max.	43.28–46.86	21.75–28.98	42.63–45.55	^#^F_(2,12.98)_ = 202.954^*^, *p* < 0.001^*^	20.67 (17.83–23.51), *p* = < 0.001^*^	0.79 (-0.67–2.25), *p* = 0.355	19.87 (17.06–22.69), *p* = < 0.001^*^	0.970 (0.931 − 0.980)
Mean ± SD. (SE)	44.95 ± 1.18 (0.42)	24.29^#@^ ± 2.66 (0.94)	44.16^$^ ± 1.04 (0.37)					
95% C.I. of mean	43.96–45.94	22.06–26.51	43.29–45.03					
Median (IQR)	44.85 (44.05–45.84)	23.83 (21.93–26.02)	44.16 (43.39–45.00)					
MD (95% C.I)	0.13 (-1.00–1.27)	16.39 (14.13–18.65)	0.34 (-0.79–1.46)					
t, p_0_	t = 0.253,p_0_ = 0.804	t = 16.555^*^,p_0_ < 0.001^*^	t = 0.645,p_0_ = 0.530					

*n* = 8 rats

*IQR* Inter quartile range, *SD* Standard deviation, *SE* Standard error of mean, *MD* Mean difference (Higher – Lower), *CI* Confidence interval, *df* degree of freedom

t: independent samples student t-test

F: F for One way ANOVA test, Pairwise comparison bet. each 2 groups was done using Post Hoc Test (adjusted Bonferroni)

p: *p* value for comparing between the three studied groups

p0: *p* value for student t-test for comparing between One month and Two month in each group

#F: Welch ANOVA test, Pairwise comparison bet. each 2 groups was done using Post Hoc Test (Games-Howell)

*: Statistically significant at *p* ≤ 0.05

#: Significant with Sham , $: Significant with OVX, @: Significant between One month and Two mont

Significant difference (*p* < 0.001) was detected among the three studied groups´ area of the connective tissue papillae, group I was (1242.5 ± 25.93, 1220.8-1264.2 µm^2^), (1245.3 ± 22.95, 1226.1-1264.5 µm^2^) at one month and two month post injection respectively, for group II was (984.4 ± 40.51, 950.5-1018.2 µm^2^), (316.4 ± 122.7, 213.8-418.9 µm^2^) at one month and two month post injection respectively, and group III was (1221.8 ± 15.04, 1209.3-1234.4 µm^2^), (1232.6 ± 12.91, 1221.8-1243.4 µm^2^) at one month and two month post injection respectively. Post hoc analysis utilizing adjusted Bonferroni test at one month post injection and Games-Howell test at two month post injection revealed that group II has decreased area compared to group I (MD = 258.1 µm^2^, 95% CI: 221.5-294.8, *p* = < 0.001), (MD = 928.9 µm^2^, 95%CI: 801.0-1056.8, *p* = < 0.001) at one month, two month post injection respectively. Furthermore, group II has significantly decreased area compared to group III at one month (MD = 237.5 µm^2^, 95% CI: 200.8-274.1, *p* = < 0.001), and at two-month (MD = 916.2 µm^2^, 95%CI: 788.4–1044.0, *p* = < 0.001) post injection. The effect size at one month and two-month post injection was 0.949(0.882–0.965) and 0.916.2(788.4–1044.0), respectively (Table 3, Fig. 6g).

**Table 3:** Comparison between the investigated groups in relation to the area of connective tissue papillae in one- and two- months post treatment

Area (µm^2^)	Sham (*n* = 8)	OVX (*n* = 8)	OVX + MSC (*n* = 8)	Test of Sig. (df), *p*	Post Hoc Test			Effect Size
					Sham vs. OVX MD (95% C.I), *p*	Sham vs. OVX+ MSC MD (95% C.I), *p*	OVX vs. OVX+ MSC MD (95% C.I), *p*	η² (95% C.I)
One month								
Min. – Max.	1202.5–1277.3	931.1–1070.5	1208.5–1250.2	F_(2,21)_ = 194.438^*^, *p* < 0.001^*^	258.1(221.5–294.8), *p* = < 0.001^*^	20.67(-16.0–57.34), *p* = 0.510	237.5(200.8–274.1), *p* = < 0.001^*^	0.949 (0.882–0.965)
Mean ± SD. (SE)	1242.5 ± 25.93(9.17)	984.4^#^±40.51(14.32)	1221.8^$^±15.04(5.32)					
95% C.I. of mean	1220.8–1264.2	950.5–1018.2	1209.3–1234.4					
Median (IQR)	1244.3 (1221.8–1264.1)	984.9 (960.8–991.0)	1216.4 (1211.3–1230.3)					
Two month								
Min. – Max.	1207.5–1278.7	141.3–527.3	1217.9–1254.7	^#^F_(2,11.49)_ = 211.373^*^, *p* < 0.001^*^	928.9(801.0–1056.8), *p* = < 0.001^*^	12.71 (-12.43–37.85), *p* = 0.391	916.2(788.4–1044.0 ), *p* = < 0.001^*^	0.976 (0.945–0.984)
Mean ± SD. (SE)	1245.3 ± 22.95(8.12)	316.4^#@^±122.7(43.37)	1232.6^$^ ± 12.91(4.57)					
95% C.I. of mean	1226.1–1264.5	213.8–418.9	1221.8–1243.4					
Median (IQR)	1245.3 (1230.1–1262.6)	291.2 (238.5–401.5)	1227.9 (1223.2–1242.9)					
MD (95% C.I)	2.77 (-23.49–29.03)	667.99(563.7–772.2)	10.73 (-4.30–25.76)					
t, p_0_	t = 0.226,p_0_ = 0.824	t = 14.624^*^,p_0_ < 0.001^*^	t = 1.531,p_0_ = 0.148					

*n* = 8 rats

*IQR* Inter quartile range, *SD* Standard deviation, *SE* Standard error of mean, *MD* Mean difference (Higher – Lower), *CI* Confidence interval, *df* degree of freedom

t: independent samples student t-test

F: F for One way ANOVA test, Pairwise comparison bet. each 2 groups was done using Post Hoc Test (adjusted Bonferroni)

p: *p* value for comparing between the three studied groups

p0: *p* value for student t-test for comparing between One month and Two month in each group

#F: Welch ANOVA test, Pairwise comparison bet. each 2 groups was done using Post Hoc Test (Games-Howell)

*: Statistically significant at *p* ≤ 0.05

#: Significant with Sham, $: Significant with OVX, @: Significant between One month and Two month

The three studied groups revealed significant difference (*p* < 0.001) in the *collagen area%* of the connective tissue papillae, group I was (70.03 ± 0.1.87, 68.47–71.60%), (70.29 ± 1.92, 68.68–71.90%) at one month and two month post injection respectively, for group II was (43.64 ± 3.02, 41.12–46.17%), (32.02 ± 4.63, 28.15–35.90) at one month and two month post injection respectively, and group III was (67.80 ± 3.01, 65.29–70.3%), (70.64 ± 3.25, 67.92–73.35%) at one month and two month post injection respectively. Post hoc analysis utilizing adjusted Bonferroni test at one month post injection and Games-Howell test at two month post injection revealed that group II has decreased collagen area% compared to group I (MD = 26.39%, 95% CI: 23.01–29.78, *p* = < 0.001), (MD = 38.27%, 95%CI:33.92–42.62, *p* = < 0.001) at one month, two month post injection respectively, And group II has significantly decreased collagen area% compared to group III at one month (MD = 24.16, 95% CI: 20.78–27.55, *p* = < 0.001), and at two month (MD = 38.61, 95%CI: 34.26–42.96, *p* = < 0.001) post injection. The effect size at one month and two-month post injection were 0.958(0.903–0.971) and 0.96 (0.929–0.979) (Table 4, Fig. 6i).

**Table 4:** Comparison between the investigated groups in relation to the collagen area% of connective tissue papillae in one- and two- months post treatment

Collagen area%	Sham (*n* = 8)	OVX (*n* = 8)	OVX + MSC (*n* = 8)	F (df), *p*	Post Hoc Test			Effect Size
					Sham vs. OVX MD (95% C.I), *p*	Sham vs. OVX+ MSC MD (95% C.I), *p*	OVX vs. OVX+ MSC MD (95% C.I), *p*	η² (95% C.I)
One month								
Min. – Max.	67.81–72.88	39.72–48.65	64.76–74.28	F_(2,21)_ = 237.472^*^, *p* < 0.001^*^	26.39 (23.01–29.78), *p* = < 0.001^*^	2.23 (-1.16–5.61), *p* = 0.335	24.16 (20.78–27.55), *p* = < 0.001^*^	0.958 (0.903–0.971)
Mean ± SD. (SE)	70.03 ± 1.87 (0.66)	43.64^#^ ± 3.02 (1.07)	67.80^$^ ± 3.01 (1.06)					
95% C.I. of mean	68.47–71.60	41.12–46.17	65.29–70.32					
Median (IQR)	69.20 (68.76–71.82)	43.63 (41.08–45.67)	66.97 (66.11–68.62)					
Two month								
Min. – Max.	67.92–73.34	24.46–38.15	64.04–73.92	F_(2,21)_ = 331.207^*^, *p* < 0.001^*^	38.27 (33.92–42.62), *p* = < 0.001^*^	0.34 (-4.00–4.69), *p* = 1.000	38.61 (34.26–42.96), *p* = < 0.001^*^	0.969 (0.929–0.979)
Mean ± SD. (SE)	70.29 ± 1.92 (0.68)	32.02^#@^ ± 4.63 (1.64)	70.64^$^ ± 3.25 (1.15)					
95% C.I. of mean	68.68–71.90	28.15–35.90	67.92–73.35					
Median (IQR)	69.60 (68.96–71.97)	32.22 (28.67–35.90)	70.75 (69.37–73.44)					
MD (95% C.I)	0.26 (-1.78–2.30)	11.62 (7.43–15.81)	2.83 (-0.52–6.19)					
t, p_0_	t = 0.274,p_0_ = 0.788	t = 5.946^*^,p_0_ < 0.001^*^	t = 1.809,p_0_ = 0.092					

*n* = 8 rats

*IQR* Inter quartile range, *SD* Standard deviation, *SE* Standard error of mean, *MD* Mean difference (Higher – Lower), *CI* Confidence interval, *df* degree of freedom

t: independent samples student t-test

F: F for One way ANOVA test, Pairwise comparison bet. each 2 groups was done using Post Hoc Test (adjusted Bonferroni)

p: *p* value for comparing between the three studied groups

p0: *p* value for student t-test for comparing between One month and Two month in each group

*: Statistically significant at *p* ≤ 0.05

#: Significant with Sham, $: Significant with OVX, @: Significant between One month and Two month

Assessment of *the keratin thickness* of the inferior lingual epithelium (8 rats per subgroup, *n* = 8 rats) of the three studied groups revealed significant difference (*p* < 0.001), group I showed (19.12 ± 2.66, 16.89–21.34 μm), (19.87 ± 1.56, 18.56–21.17 μm) at one month and two month post injection respectively, for group II was (10.28 ± 0.94, 9.49–11.06 μm), (8.18 ± 1.50, 6.93–9.43 μm) at one month and two month post injection respectively, and group III was (17.51 ± 1.06, 16.62–18.39 μm), (18.82 ± 1.62, 17.47–20.18 μm) at one month and two month post injection respectively. Post hoc analysis utilizing adjusted Bonferroni test at one month post injection and Games-Howell test at two month post injection revealed that group II has decreased keratin thickness compared to group I (MD = 8.84 μm, 95% CI: 6.04–11.64, *p* = < 0.001), (MD = 11.69 μm, 95%CI: 9.72–13.65, *p* = < 0.001) at one month, two month post injection respectively. And group II has significantly decreased keratin thickness compared to group III at one month (MD = 7.23 μm, 95% CI: 5.92–8.54, *p* = < 0.001), and at two-month (MD = 10.64 μm, 95%CI: 8.68–12.61, *p* = < 0.001) post injection. The effect size at one month post injection was 0.848 (0.667–0.898) and two-month post injection was 0.929 (0.839–0.952) (Table 5, Figs. 5h-m, 6j).

**Table 5:** Comparison between the three studied groups according to Keratin thickness of the inferior surface of the tongue epithelium (µm)

Keratin thickness (µm)	Sham (*n* = 8)	OVX (*n* = 8)	OVX + MSC (*n* = 8)	Test of Sig. (df), *p*	Post Hoc Test			Effect Size
					Sham vs. OVX MD (95% C.I), *p*	Sham vs. OVX+ MSC MD (95% C.I), *p*	OVX vs. OVX+ MSC MD (95% C.I), *p*	η² (95% C.I)
One month								
Min. – Max.	14.85–22.81	8.88–11.74	16.40–19.22	^#^F_(2,12.90)_ = 115.826^*^, *p* < 0.001^*^	8.84 (6.04–11.64), *p* = < 0.001^*^	1.61 (-1.21–4.42), *p* = 0.298	7.23 (5.92–8.54), *p* = < 0.001^*^	0.848 (0.667–0.898)
Mean ± SD. (SE)	19.12 ± 2.66 (0.94)	10.28^#^ ± 0.94 (0.33)	17.51^$^ ± 1.06 (0.37)					
95% C.I. of mean	16.89–21.34	9.49–11.06	16.62–18.39					
Median (IQR)	19.42 (17.28–20.93)	10.01 (9.72–11.06)	17.34 (16.51–18.36)					
Two month								
Min. – Max.	16.98–21.37	6.48–10.30	15.82–20.64	F_(2,21)_ = 137.645^*^, *p* < 0.001^*^	11.69 (9.72–13.65), *p* = < 0.001^*^	1.04 (-0.92–3.01), *p* = 0.586	10.64 (8.68–12.61), *p* = < 0.001^*^	0.929 (0.839–0.952)
Mean ± SD. (SE)	19.87 ± 1.56 (0.55)	8.18^#@^ ± 1.50 (0.53)	18.82^$^ ± 1.62 (0.57)					
95% C.I. of mean	18.56–21.17	6.93–9.43	17.47–20.18					
Median (IQR)	20.15 (19.03–21.11)	7.68 (7.0–9.65)	19.25 (17.99–19.83)					
MD (95% C.I)	0.75 (-1.58–3.09)	2.09 (0.75–3.43)	1.32 (-0.15–2.78)					
t, p_0_	t = 0.691,p_0_ = 0.501	t = 3.353^*^,p_0_ = 0.005^*^	t = 1.929,p_0_ = 0.074					

*n* = 8 rats

*IQR* Inter quartile range, *SD* Standard deviation, *SE* Standard error of mean, *MD* Mean difference (Higher – Lower), *CI* Confidence interval, *df* degree of freedom

t: independent samples student t-test

F: F for One way ANOVA test, Pairwise comparison bet. each 2 groups was done using Post Hoc Test (adjusted Bonferroni)

p: *p* value for comparing between the three studied groups

p0: *p* value for student t-test for comparing between One month and Two month in each group

#F: Welch ANOVA test, Pairwise comparison bet. each 2 groups was done using Post Hoc Test (Games-Howell)

*: Statistically significant at *p* ≤ 0.05

#: Significant with Sham, $: Significant with OVX, @: Significant between One month and Two month

## Discussion

Menopause is characterized by significant decrease in ovarian hormones and is associated with profound impact on the oral health [9, 20, 21, 54]. Ovariectomized rat is a commonly utilized animal model to evaluate postmenopausal complications [10–12, 21, 34]. In the present work, to evaluate the efficacy of BM-MSCs in ameliorating the menopausal changes in lingual filiform and fungiform papillae, ovariectomized rats were utilized. In present study, to confirm the estrogen deficiency in the ovariectomized animals, the BMD was evaluated as low BMD is associated with estrogen deficiency in menopause [55–57]. Our results revealed decreased BMD in the ovariectomized rats compared to their controls. Our results are in compliance with the findings of previous studies which demonstrated low BMD in ovariectomized animals confirming estrogen deficiency in postmenopausal animals [57, 58].

In this work, the tongue was the tissue of inquiry since it is highly affected by burning tongue feeling in menopause [9, 59]. Instead of the systemic route, local administration of BM-MSCs was selected, as local transplantation of BM-MSCs has better therapeutic results in the irradiated [60] and diabetic [28, 61] rat tongue, since it ensures homing of MSCs [62] and, unlike the systemic administration, permits more labelled MSCs to reach the tongue [63].

Our SEM results revealed abnormal surface structure of the filiform and fungiform papillae of the ovariectomized rats including roughened, cracked and desquamated overlying keratin of the filiform papillae and deformity and atrophy of the taste bud in the fungiform papillae. Our results go hand in hand with the data obtained by Niu et al. [21], who explained that changes in the filiform papillae may result from the partial loss of the associated small nerve ending. This loss decreases the ability of the lingual mucosa to tolerate the mechanical stimulants [21]. In addition, Niu et al., 2021 speculated that low estrogen level may result in deformation of the chorda tympani nerve, which provide the taste supply to the taste buds, or may cause upregulation of the transient ion channels found at the nerve endings, causing alterations in the architecture and function of the taste papillae [21].

Our results revealed irregular papillary architecture with signs of cellular degeneration recognized in the tongue of ovariectomized rats. Some cells were enlarged or had indistinct boundaries, while others represented pyknotic or degenerated nuclei. Basal cells showed areas of increased cellularity or flattening. Thin and detached keratin threads were also seen in some areas. In addition, the ovariectomized groups depicted wide vacant degenerated connective tissue areas. Further, the lamina propria displayed sparse collagen fibers and inflammatory cell infiltrate. Our results are in accordance with the previous results in variable organs in ovariectomized animals [4, 64]. These degenerative changes could result from oxidative stress brought on by estrogen deprivation in the ovariectomized animals. Oxidative stress can trigger cellular injury by production of massive amounts of free radicals resulting in damage of the DNA double helical structure [65]. Generation of reactive oxygen species is also linked with the decline of mitochondrial antioxidant factors [66, 67]. Therefore, cellular protein destruction may be caused by oxidative stress arising from disparity between the excess production of reactive oxygen species and their neutralization by inherent antioxidant protective mechanisms [4]. In addition, Xie et al. clarified that ovariectomy causes overexpression of inducible nitric oxide synthase plus tumor necrosis factor- α genes, thereby starting an inflammatory response [68]. In addition, our results are in accordance with the results of Chen et al.; El-Badawy et al.; Yassa et al. who reported darkly stained or completely degenerated nuclei in acinar salivary glands, liver and adrenal cortex cells of ovariectomized rats [10, 69, 70]. Hypercellularity and enlarged hepatocytes were also detected in untreated menopausal rats [71].

In this study, epithelial ridges in the ovariectomized group appeared short and ill-defined with apparent loss of some connective tissue papillae. Our data are in line with the findings of Seko et al. and Niu et al. who demonstrated thinning of the lingual epithelium in the tongue of ovariectomized animals [20, 21]. The authors suggested that these findings could be attributed to estrogen deficiency. Niu et al. explained that estrogen plays a crucial role in promoting proliferation and this role may be promoted via epidermal growth factor effect on the basal membrane of the lingual epithelium [21].

In this work, the basal cells of the lingual epithelium of the ovariectomized rats appeared blurred with disturbed architecture and areas of detachment, which goes in line with the findings of Niu et al. [21]. The authors explained that estrogen deficiency could influence the desmosomal junction between the basal cells in the dorsal lingual epithelium and their ultrastructure as well. In addition, the authors further clarified that estrogen may influence the laminin in the basement membrane; therefore, estrogen deficiency may cause structural injury and indistinct basement membrane [21, 72].

In this study, fungiform papillary deformity and atrophy of its taste buds were identified in the ovariectomized rats. Niu et al. speculated that low levels of estrogen in menopausal woman might result in anomalies of chorda tympani nerve or elevated transitory ion channel expression on nerve terminals, evoking variations within the fungiform papillae and taste buds’ structure [21]. Furthermore, Rizali et al. proposed that 6-n-propylthiouracil receptors located in the taste buds are sensitive to estrogen. This association led to a decline in the taste bud cells’ proliferative activity following induced menopausal condition [73].

In our investigation, BM-MSC treated ovariectomized rat tongues dissected one-month post-injection, showed ameliorative effect on the filiform and fungiform papilla structure. The tongue depicted normal epithelial strata with focal areas of altered basal cells architecture and keratin detachment and near normal configuration of the connective tissue papillae. Well defined regenerated taste buds were also manifested. Our SEM results ensured these findings representing almost uniform filiform papillary orientation with minimal desquamated keratin fragments. Obviously unblocked taste pore was also identified on the summit of the fungiform papilla. In addition, the lamina propria in BM-MSCs treated groups revealed apparently fewer areas of degeneration, more densely organized collagen fibers with less inflammatory cell infiltration when compared to the ovariectomized ones. Previous investigations revealed similar findings with minor areas of papillary destruction in BM-MSCs treated tongue of diabetic [28] and hypothyroid rats [48]. Likewise, restoration of the regular salivary gland structure in ovariectomized [10–12], irradiated [29, 31, 74] and diabetic animals [75] and in the periodontium of carbimazole-treated rats [53] infused with BM-MSCs was reported.

It has been reported that the regenerative influence of MSCs can be direct and indirect. The MSCs can act directly through their ability to multilineage differentiation. They can act indirectly, through their ability to secrete various bioactive molecules including transforming growth factor-ꞵ, vascular endothelial growth factor (VEGF), insulin growth factor-1, hepatocyte growth factor and angiogenin. These molecules play pivotal anti-inflammatory, anti-apoptotic, immunomodulatory effect and role in angiogenesis. Therefore, it is reported that these molecules support the regenerative microenvironment and recruit the resident cells to repair the damaged tissue [36, 76, 77]. In addition, it has been demonstrated that angiogenesis is a crucial process in repairing the injured tissues. It is reported to be mediated via two mechanisms: the differentiation of the MSCs into pericytes, vascular smooth muscle and endothelial cells, and the second mechanism is through secretion of various angiogenic factors by the MSCs such as VEGF. It has been shown that the activation of the VEGF signaling via GSKꞵ/ꞵ-catenin and PI3K/AKT cascades leads to vascular remodeling and ultimately tissue formation [77, 78]. In addition, Fan et al. assumed that MSCs may bring about their effect by secreting exosomes containing reparative peptides/proteins, mRNA, and microRNA [79].Currently, cell-based therapy utilizing isolated living cells to restore the structure and function of the damaged tissues i.e. salivary glands has approached large step forward [80]. In irradiated patients, intraglandular administration of MScs improved the unstimulated salivary flow, increased the ductal and acinar areas and decreased the fibrosis after 4 months [81, 82]. And MSCs utilization in clinical trials in treating premature ovarian failure has been carried out using intraovarian transplantation. However, research is going on to increase the safety and efficacy of MSCs-based therapy and to minimize the side effects [77].

Although not statistically significant, mild amelioration in the histological picture was observed in ovariectomized rats treated with stem cells for two months than one month. The amelioration in the histological structure is in agreement with Musiał-Wysocka et al. and Zare et al. [76, 83] who clarified that BM-MSCs transplantation showed improvement of some pathological features in a time-dependent manner.

A limitation of the current work is the lack of molecular investigations explaining the mechanistic pathways underlying the histopathological alteration in the ovariectomized rat tongues. Therefore, further studies are required to assess the mechanistic pathways acting on the altered lingual mucosa in ovariectomized rats to uncover possible cause, including the epithelial cell marker protein expression change in the filiform and fungiform papillae is recommended, for proper planning of possible therapeutic potentials. In addition, future studies evaluating the effect of the administration of BMMSCs compared to estradiol and/or naturally derived herbs are recommended. Further investigations are required to validate findings of the present study.

## Conclusions

We concluded that transplanted BM-MSCs ameliorate the altered histological and surface structure of the filiform and fungiform papillae of the lingual mucosa of ovariectomized rats, however, the mechanism underlying this effect needs further investigation. Our findings may suggest a promising therapeutic option in promoting the recovery of the lingual mucosal changes induced by ovariectomy.

## Data Availability

Data are provided within the manuscript or Supplementary Information files and are available from the corresponding author maha.elshahawy@den.kfs.edu.eg upon a reasonable request.

## Abbreviations

BMD: Bone mineral density
BM-MSCs: Bone marrow mesenchymal stem cells
DEXA: Dual-energy X-ray absorptiometry
H&E: Hematoxylin and Eosin
MSCs: Mesenchymal stem cells
SEM: Scanning electron microscope
VEGF: Vascular endothelial growth factor
